# Regional Comparison of Nitrogen Export to Japanese Forest Streams

**DOI:** 10.1100/tsw.2001.371

**Published:** 2001-11-22

**Authors:** Hideaki Shibata, Koichiro Kuraji, Hiroto Toda, Kaichiro Sasa

**Affiliations:** Northern Forestry Research and Development Office, Field Science Center for Northern Biosphere, Hokkaido, Nayoro, Japan

**Keywords:** nitrogen, stream chemistry, biogeochemistry, regional comparison, nitrogen saturation, Japan, forested watershed

## Abstract

Nitrogen (N) emissions in Asian countries are predicted to increase over the next several decades. An understanding of the mechanisms that control temporal and spatial fluctuation of N export to forest streams is important not only to quantify critical loads of N, N saturation status, and soil acidification N dynamics and budgets in Japanese forested watersheds is not clear due to the lack of regional comparative studies on stream N chemistry. To address the lack of comparative studies, we measured inorganic N (nitrate and ammonium) concentrations from June 2000 to May 2001 in streams in 18 experimental forests located throughout the Japanese archipelago and belonging to the Japanese Union of University Forests. N concentrations in stream water during base flow and high flow periods were monitored, and N mineralization potential in soil was measured using batch incubation experiments. Higher nitrate concentrations in stream water were present in central Japan, an area that receives high rates of atmospheric N deposition. In northern Japan, snowmelt resulted in increased nitrate concentrations in stream water. The potential net N mineralization rate was higher in surface soil than in subsurface soil, and the high potential for N mineralization in the surface soil partly contributed to the increase in nitrate concentration in stream water during a storm event. Regional differences in the atmospheric N deposition and seasonality of precipitation and high discharge are principal controls on the concentrations and variations of nitrates in stream water in forested watersheds of Japan.

### INTRODUCTION

Development of industrial and agricultural activities during the 20th century has increased atmospheric deposition of nitrogen (N) worldwide. Although N is an essential nutrient for biota, an excess of N deposition can cause degradation of natural ecosystems and eutrophication of freshwater and coastal and marine waters[1]. Numerous studies conducted over the past 2 decades have shown the significant contribution of atmospheric deposition of N to nitrate concentrations in stream water, especially in northern Europe[2,3,4]. N emissions in Asian countries are predicted to increase significantly over the next several decades[5].

Predictions of future effects of atmospheric N deposition in Asia are dependent on assessments of the current status of nitrate in stream water in Asia; however, few regional surveys[6,7,8] have been performed due to a lack of regional monitoring projects for stream biogeochemistry in this region. The geology (dominated by volcanic rocks) and climate (monsoon) in eastern Asia is very different from that in the U.S. and northern Europe[9,10]. The unique regional environmental factors may result in different characteristics of N biogeochemistry in forested watersheds of eastern Asia than in northeastern America and Europe[10,11]. In this study, we compared the nitrate concentrations in stream water in 18 Japanese forested watersheds located throughout the Japanese archipelago. The main objective of this study was to clarify the factors contributing to regional and temporal fluctuations in nitrate concentrations in Japanese forest streams. This study was conducted as part of a joint monitoring project by the Japanese Union of University Forests.

### METHODS

#### Study Sites

Eighteen watersheds located throughout the Japanese archipelago were studied (Fig. 1). General information on each watershed is given in Table 1. The basin area, annual precipitation, and annual mean temperature in each watershed ranged from 0.008 to 4.8 $km^2$, from 770 to 3010 mm $year^{-1}$, and from 2.5 to 16$^oC$, respectively. Bedrock underlying the watersheds includes sandstone, slate, biotite, granite, diorite, shale, andesite, tuff breccia, mudstone, quartzite, granodiorite porphyry, deposited pumice, chert, and tuff. Five of the watersheds contain plantations of Japanese cedar (*Criptomeria japonica*) and Hinoki cypress (*Chamaecyparis obtusa*), which are common species for commercial wood in Japan. Nine of the watersheds are covered by natural forests of deciduous broad-leaved, evergreen broad-leaved, and coniferous evergreen species. Some of the "natural" forests were second-growth forests. Seven of the watersheds (HK-M3, NG-SR, NK-SR, TB-KH, TK-DS, TK-FA, and MY-7N) are gauged to measure the stream discharge, although the annual runoff during the studied period was not available in most sites due to data limitation.

#### Sampling Method

Stream water was collected at the outlet of each watershed every other month during the base flow period from June 2000 to May 2001. Some streams were sampled during extended high flow periods, especially during the early spring when snowmelt occurs in northern Japan (HK-M3 and KS-KW). At KS-KW, stream water was collected monthly. Water was collected in polyethylene bottles that were washed with ultrapure water before sampling. The sampled water was immediately transported to the laboratory of the Northern Forestry Research and Development Office, Field Science Center for Northern Biosphere, Hokkaido University. The temperature of the samples was kept below 4$^oC$

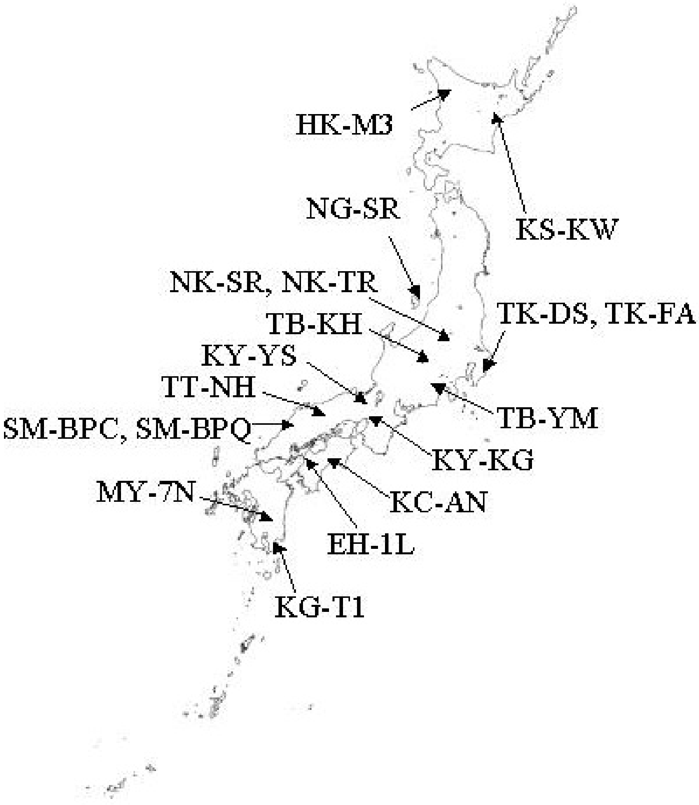

**FIGURE 1**. Locations of the watershed study sites. See Table 1 for site codes.

**TABLE 1**
**General Information of Studied Watershed**

| Code | Location | University[1)] | Experimental Forest | Basin area $km^2$ | Annual precipitation mm $y^{-1}$ | Annual mean temperature °C | Bedrock[2)] | Vegetation[3,4)] | Altitude m.a.s.l |
|---|---|---|---|---|---|---|---|---|---|
| HK-M3 | 44° 21'N 142° 15'E | Hokkaido Univ. | Uryu | 0.2 | 1375 | 2.5 | Ad | MNR | 290–310 |
| KS- KW | 43° 19'N 143° 31'E | Kyushu Univ. | Hokkaido | 4.8 | 770 | 6 | Tf, Ss, Sh | BNR | 230–510 |
| NG - SR | 38° 12'N 138° 26'E | Niigata Univ. | Sado | 0.073 | 1563 | 8.9 | Ad | MNR | 653–822 |
| NK-SR | 36° 33'N 139° 20'E | TUAT | Ohyasan | 0.018 | 1547 | 10 | Ss, Sl | PL (Cj, Co) | 780–870 |
| NK-TR | 36° 33'N 139° 20'E | TUAT | Ohyasan | 0.013 | 1547 | 10 | Ss, Sl | PL (Cj, Co) | 790–870 |
| TB-KH | 35° 55'N 138° 30'E | Tsukuba Univ. | Kawakami | 0.39 | 1150 | 6.2 | Ad | BNR, PL(Ll) | 1440–1786 |
| TB-YM | 35° 20'N 138° 13'E | Tsukuba Univ. | Ikawa | 1.1 | 2766 | 9.4 | Ss, Sh | MNR | 1160–2167 |
| KY- YS | 35° 19'N 135° 43'E | Kyoto Univ. | Asyu | 0.08 | 2375 | 14 | Ct, Ss | MNR | 490–740 |
| TK- DS | 35° 12'N 140° 09'E | The Univ. of Tokyo | Chiba | 0.014 | 2425 | 14.1 | Ss, Ms | MNR | 152–253 |
| TK- FA | 35° 12'N 140° 06'E | The Univ. of Tokyo | Chiba | 0.008 | 2155 | 14 | Ss, Ms | PL (Cj, Co) | 126–230 |
| SM-BPC | 35° 09'N 132° 37'E | Shimane Univ. | Sanbe | 0.15 | 2471 | 12.1 | Bt, Gn, Dr | PL (Cj, Co) | 374–582 |
| SM-BPQ | 35° 09'N 132° 37'E | Shimane Univ. | Sanbe | 0.17 | 2471 | 12.1 | Bt, Gn, Dr | BNR | 372–508 |
| KY- KG | 35° 04'N 135° 46'E | Kyoto Univ. | Kamigamo | 0.05 | 1679 | 15.6 | Ss, Sl | BNR | 100–225 |
| TT - NH | 35° 01'N 133° 35'E | Tottori Univ. | Hiruzen | 1.6 | 2164 | 12.6 | Tb | BNR | 630–859 |
| EH- 1L | 33° 55'N 132° 54'E | Ehime Univ. | Ehime | 0.048 | 1945 | 12.2 | Gp | PL (Cj, Co), BNR | 540–700 |
| KC- AN | 33° 42'N 133° 36'E | Kochi Univ. | Minekita | 4.4 | 2500 | 12 | Qt | PL (Cj, Co), BNR | 510–1050 |
| MY- 7N | 31° 51'N 131° 18'E | Miyazaki Univ. | Tano | 0.03 | 2800 | 16 | Ss, Sh | PL (Cj, Co) | 170–220 |
| KG- T1 | 31° 32'N 130° 46'E | Kagoshima Univ. | Takakuma | 0.43 | 3010 | 14 | Ss, Dp | PL (Cj, Co), ENR | 520–678 |

1) TUAT: Tokyo University of Agriculture and Technology
2) Ss: Sandstone, Sl: Slate, Bt Biotite, Gn: Granite, Dr: Diorite, Sh: Shale, Ad: Andesite, Tb: Tuff Breccia, Md: Mudstone, Qt: Quartzite, Gp: Granodiorite porphyry, Dp: Deposited pumice, Ct: Chert, Tf: Tuff
3) PL: Plantation, DNR: Natural deciduous-broad leaved forest, MNR; Mixed natural forest, ENR: Natural evergreen-broad leaved forest
4) Cj: Criptomeria japonica, Co: Chamaecyparis obtusa, Ll: Larix leptolepis during transportation. In the laboratory, each sample was filtered using a GF/F filter that had been heated in advance for 3 h at 450°C. Nitrate and ammonium concentrations in the stream water were measured by ion chromatography (Dionex Co. Ltd, DX-500) after filtering through a membrane filter (pore size: 0.2 µm). The analytical detection limits for nitrate and ammonium concentration of the chromatography were 0.00056 and 0.0019 mg N $l^{-1}$, respectively. All samples collected every other month were analyzed by the same analyzer using the same analytical protocol in order to eliminate possible errors due to different analytical processes used in the laboratories. The mean concentration of nitrate in samples collected every other month was calculated as the arithmetic average, because stream discharge data were not available for all streams. Precipitation amount and air temperature were measured continuously at some watersheds.

### Potential Nitrogen Mineralization of Soil

Surface soil was collected in ten watersheds to determine the net potential mineralization of N in the soil. Soil was collected at three spots located in an unsaturated area at the lower part of a slope in each watershed. After the removal of forest floor and litter (O horizon), mineral soil was collected at depths of 0 to 10 cm and 10 to 20 cm and was mixed and sieved using a filter (4-mm mesh) to remove coarse gravels, roots, and litter. The total amount of soil collected at each depth was about 1 kg. The sampled soil was incubated in glass beakers for 6 weeks at 25°C in an incubator in the laboratory of Tokyo University of Agriculture and Technology. The water content of the incubated soil was kept at about 60% of maximum water capacity[12]. The N (nitrate and ammonium) contents of the soil were determined before and after the incubation[12]. The soil was extracted using 2 N-KCl (1 [soil]:5 [KCl]). Nitrate and ammonium concentrations in the KCl extract were measured using an FIA analyzer (Sanuki Co., Ltd., Tokyo, Japan). The potential net N mineralization rate was calculated from the difference in N contents before and after the incubation. Total carbon (C) and N content of the soil was measured before the incubation using a CN analyzer (Yanako Co., Ltd., Tokyo, Japan).

### Intensive Observation During a Storm Event

Temporal fluctuations in discharge and nitrate concentrations in stream water during storm events were monitored at stream gauges in three watersheds (NG-SR, TK-FA, and HK-M3). The water level of the stream at a weir was recorded at time intervals of 10 min to 1 h using a data logger. The recorded water level (measured in cm) was converted into stream discharge ($m^3$ $s^{-1}$) using an empirical equation that describes the relationship between water table and discharge. Stream water was collected at each weir at intervals of 10 min to 1 h using an autosampler (the interval was different among the sites), and the nitrate concentrations in the water samples were measured in each laboratory (Niigata University, The University of Tokyo and Hokkaido University) using ion chromatography after filtering.

## RESULTS AND DISCUSSION

### Regional Differences in Nitrate Concentrations in Stream Water

There were large regional differences in mean nitrate concentrations of stream water sampled every other month (Fig. 2). Mean nitrate concentrations of stream water collected in NK-SR and NK-TR were significantly higher than those in stream water collected in other watersheds (ANOVA, $p < 0.001$). The nitrate concentrations in stream water collected in KS-KW, NG-SR, TK-DS, TK-FA, SM-BPC, and EH-1L also tended to be higher than those in stream water collected at the other watersheds. Ammonium concentrations in each sample were very low compared to nitrate concentrations in all streams and were below the analytical detection limit for some samples (data not shown). The mean nitrate concentration in stream water was not related significantly to basin area, the lowest or highest altitude of the basin, annual precipitation, or annual mean temperature (using linear regression).

The net N mineralization rate of soil collected from a depth of 0 to 10 cm was higher than that of soil collected from a depth of 10 to 20 cm in all watersheds (Fig. 3). In most of the soils collected from the surface and subsurface, most of the nitrate in the incubated samples originated from nitrification, indicating that nitrification occurred rapidly with the mineralization process in these soils. Only in HK-M3 did mineralized N in incubated soils consist mostly of ammonium (at both depths). The C/N ratio of the soil ranged from 13 to 31, and soils from the northern region had higher C/N ratios. The surface soil (0- to 10-cm depth) in NG-SR, in which the mean nitrate concentration in stream water was slightly higher than that of other watersheds, had a higher rate of potential N mineralization than in other watersheds. However, the spatial difference of mean nitrate concentration in stream water (Fig. 3) was not explained significantly (using linear regression) by the C/N ratio or the net N mineralization rate of collected soil.

Fig. 4 shows the annual mean rate of atmospheric N deposition in Japan from 1993 to 1997 as a function of latitude. The data were provided by the Japanese Environment Agency and Acid Deposition and Oxidant Research Center[13]. High rates of atmospheric N deposition were observed mainly in central Japan (from 33 to 38 $^{o}$N ), where most large cities and industrial centers are located. Since high concentrations of nitrate in streams were also found in central Japan near Tokyo (Gunma Prefecture), the high flux of atmospheric N deposition appears to have contributed to the high nitrate concentrations in streams in this region. Mitchell et al.[6] reported that analysis of annual budgets using reported data showed that N concentrations in stream water from forest watersheds in central Japan were close to a state of N saturation. Although our data suggest that the regional difference of the N deposition primarily caused the regional difference of the mean concentration of nitrate in stream water during a base flow, the roles of many other factors (such as forest type, management history, and climate) on the formation of nitrate concentration in stream water are needed for a more detailed interpretation of the geographical comparison[6,7].

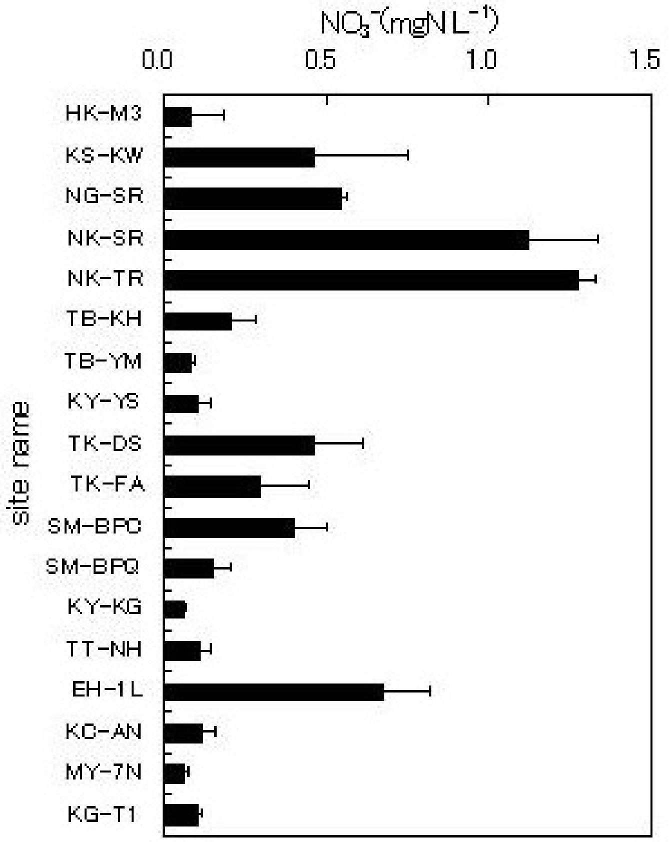

**FIGURE 2**. Mean nitrate concentrations in stream water sampled every other month from June 2000 to May 2001 in each watershed.

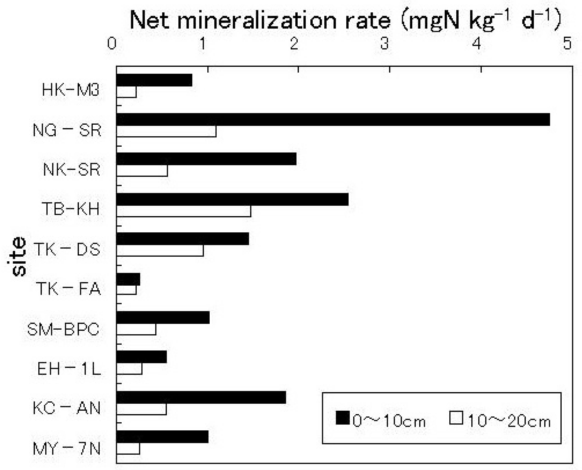

**FIGURE 3**. Net mineralization rates of N in soil (6 weeks, 25°C) in the watersheds.

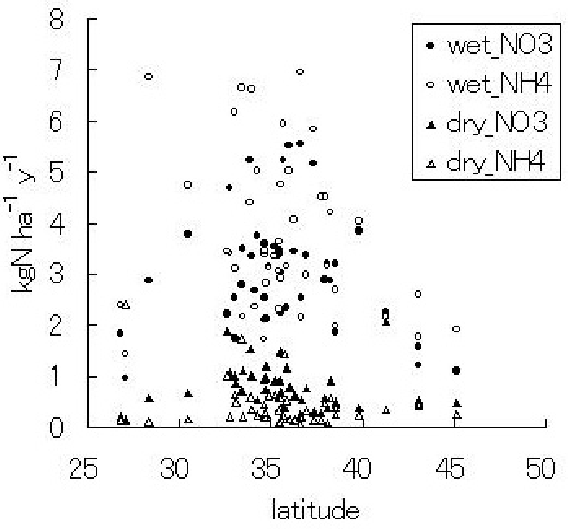

**FIGURE 4**. Annual mean atmospheric N deposition from 1993 to 1997 (Japanese Environmental Agency and Acid Deposition and Oxidant Research Center, 1999). Closed and open circles represent wet deposition of nitrate and ammonium, respectively. Closed and open triangles represent dry deposition of nitrate and ammonium, respectively.

### Seasonal Fluctuation in Nitrate Concentrations in Stream Water

Fig. 5 shows the seasonal fluctuations in monthly precipitation, monthly mean temperature, and nitrate concentration in stream water in KS-KW, NK-TR, and KY-KG. These areas cover a large geographical range, with KS-KW being in a northern island (Hokkaido) and NK-TR and KY-KG being in central and western Japan (Honshu islands). These areas also cover a large range of nitrate concentration in stream water (NK-TR: high concentration, KS-KW: medium concentration, and KY-KG: low concentration, see Fig. 2). There were large amounts of precipitation during the warm period from July to September at all three sites. Only in KS-KW did significant snowfall occur in winter, and some soil was frozen due to insufficient snowpack (data not shown). The peak flow of stream discharge due to snowmelt in KS-KW occurred from late March to April. The increase in the nitrate concentration in stream water at KS-KW in April was related to the flushing of nitrate from the soil due to melting snow. This phenomenon of increased nitrate export to streams during snowmelt has often been reported in the northeastern U.S.[4,14,15]. The increase of nitrate concentrations in stream water during snowmelt was also observed in HK-M3, which is also located in a cold and snowy region. In general, N mineralization and nitrification occur mainly during warm periods, because most microbiological processes are temperature dependent. Even though N mineralization rates are low in winter, biological up-

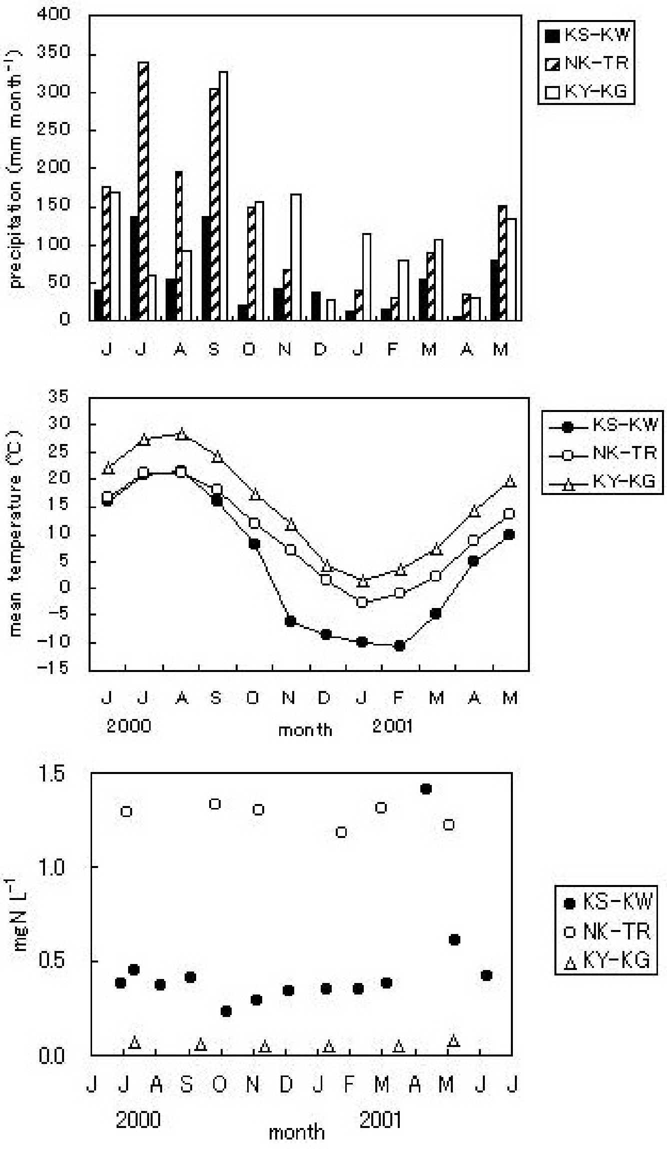

**FIGURE 5**. Monthly precipitation (top), monthly mean temperature (middle), and nitrate concentration in stream water (bottom) in KS-KW, NK-TR, and KY-KG from June 2000 to May 2001.

take of N is also low, so it appeared that more of nitrate produced under snowpack is available for leaching into the water during a snowmelt period. In this study, there was no significant seasonality of stream nitrate concentration in NK-TR and KY-KG during the year. The high concentrations of nitrate in stream water and their absence of seasonality were also observed by Ohrui and Mitchell[16] at forested catchments in Gunma Prefecture (the same region for NK-TR). They suggested that the high N output to the stream in the studied watershed in the same region of NK-TR was related to high N deposition, high N mineralization rate of soil, and low N increment of wood. A large amount of precipitation during a warm period will increase the potential of significant nitrate leaching from the surface soil to deeper soil and groundwater. Ohte et al.[11], who compared nitrate concentrations in forest streams in Japan and the U.S., pointed out that meteorological and hydrological seasonality can affect temporal fluctuations in nitrate concentrations in stream water. The nitrate concentration in stream water at KY-KG was much lower than at KS-KW and NK-TR, even though there was a relatively large amount of atmospheric N deposition at KY-KG. A surface saturated zone located near the outlet of KY-KG appears to retain some nitrate in the subsurface. Cirmo and McDonnell[17], Groffman et al.[18], and Konohira et al.[19] revealed that denitrification in the riparian saturated zone significantly contributed to removal of nitrate in the saturated ground in the near-stream zone. However, it was unclear which specific characteristics of the topography and hydrological process contributed to the N retention in the basin at KY-KG. More detailed studies will be

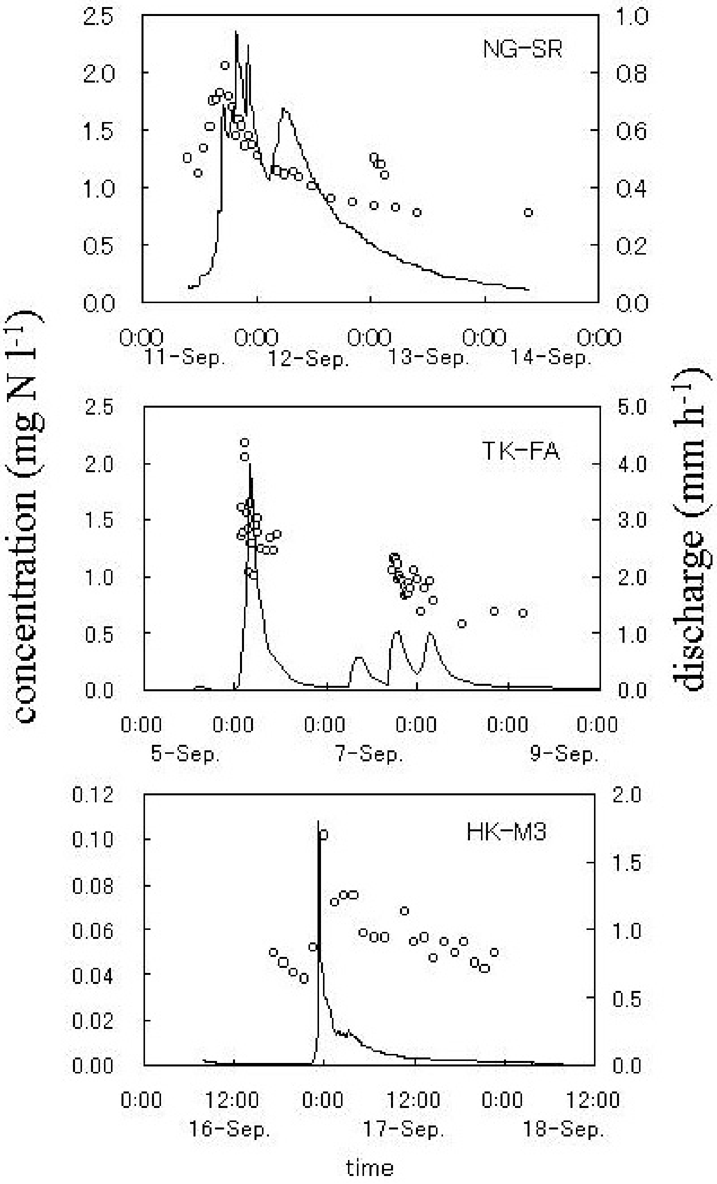

**FIGURE 6**. Temporal fluctuation in stream nitrate concentrations (open circles) and discharge (lines) during a storm in September 2000 at NG-SR (top), TK-FA (middle), and HK-M3 (bottom).

needed, including the analysis of the water flow pass and the pattern of supplies of nitrate and organic C in the subsurface water[20].

### Nitrate Export to Stream Water During Storms

The three sites for the intensive monitoring of stream water during a storm were selected by the magnitude of the nitrate concentration in stream water during a base flow: the higher (NG-SR), medium (TK-FA), and lower (HK-M3) concentrations of nitrate than other watersheds (Fig. 2). They were not coincident with the sites in Fig. 5 because of data limitations. Nitrate concentrations in stream water increased during storm events at the three watersheds that were monitored (NG-SR, TK-FA, and HK-M3, Fig. 6) compared to concentrations during low flow (Fig. 2). Nitrate concentrations in stream water during a storm reached around 2 mg N $l^{-1}$ in NG-SR and TK-FA and 0.1 mg N $l^{-1}$ in HK-M3, respectively. Since the N mineralization rate of soil tended to be higher near the surface, the increased nitrate concentrations in stream water during storms appeared to have been derived from nitrate in surface soil. Ohrui and Mitchell[21] also indicated that the soil water was a major contributor to stream water during a large storm event, although the relative contribution varied depending on the magnitude of the storm. These results support the hypothesis that a large amount of precipitation during the growing season increases the nitrate concentration in stream water during a storm. Water movement during this period also had a potential to cause the leaching of nitrate to the deeper soil and groundwater from the surface soil. This type of hydrological control has been used to explain the pattern of nitrate concentration in stream water[11,22]. More studies about the seasonality of the gradient of nitrate concentration in soil solution are needed to support the hypothesis[17,23]. Our results indicate that the linkage of hydrological processes and N dynamics in watershed is important in understanding the mechanisms that increase nitrate concentrations in stream water in this region. Since it is possible that fluctuations of nitrate concentrations in stream water are different depending on some characteristics of the storm (e.g., the magnitude of the storm, amount of precipitation, and frequency of precipitation), antecedent moisture conditions, and topographic properties of the basin[14], more comparisons will be needed to generalize the dynamics of nitrate during storms.

### CONCLUSIONS

Significant regional differences are present in nitrate concentrations in stream water at forested watersheds in Japan. High nitrate concentrations were detected in stream water collected from forest watersheds located in central Japan, probably due in part to higher rates of atmospheric N deposition in this region. Snowmelt increased nitrate concentration in stream water in the watersheds located in cold snowy regions in northern Japan. Our preliminary results suggest that the large amount of precipitation during the June to September warm season contributes to increases in nitrate concentrations in streams during storms and to leaching of nitrate from surface soil with higher potential for N mineralization to the deeper ground with lower potential for N mineralization. This downward leaching of nitrate affects nitrate concentrations in stream water during base flow.

### ACKNOWLEDGMENTS

We are grateful to Prof. T. Okano (Kyushu University, Japan), Prof. M. Nakata (Niigata University, Japan), Prof. K. Uchida and Prof. T. Ohtsuki (Tsukuba University, Japan), Prof. N. Tanaka (The University of Tokyo, Japan), Prof. N. Katagiri and Prof. T. Yamashita (Shimane University, Japan), Prof. A. Nakanishi and Prof. T. Nakashima (Kyoto University, Japan), Mr. S. Kohno (Ehime University, Japan), Prof. J. Tsukamoto (Kouchi University, Japan), Prof. K. Nogami and Prof. M. Takagi (Miyazaki University, Japan), and Prof. Y. Inokura (Kagoshima University, Japan). We are also grateful to Ms. Megumi Ozawa, Mr. Taku Hashiba, and Ms. Satomi Takaya (Hokkaido University) for their assistance in sample collection and analysis of stream water and for sharing data. We would also like to thank the technicians who conducted the joint monitoring programs in this study. We would also like to thank Dr. Douglas A. Burns, Dr. Richard P. Hooper, and two anonymous reviewers for their helpful comments on this article. This study was supported by the scientific research fund from MESSC, Japan (11356005).

| | | |
|---|---|---|
| Received: | July | 13, 2001 |
| Revised: | November | 6, 2001 |
| Accepted: | November | 9, 2001 |
| Published: | November | 22, 2001 |

### BIOSKETCHES

**Hideaki Shibata** is an Associate Professor at the Field Science Center for Northern Biosphere, Hokkaido University. He holds a Ph.D. from Hokkaido University, Japan. Dr. Shibata received progress awards from the Japanese Society of Soil Science and Plant Nutrition in April 2001for his work, “Relationship between Biogeochemical Cycling and Acidic Deposition in a Forest Ecosystem”. His research interests include biogeochemical dynamics in forest ecosystems including nitrogen, carbon, base cations, and protons. He is also interested in the linkage between the nutrient leaching and hydrological processes in the forest watershed, the effect of forest managements (harvesting, site preparation, etc.) on the carbon and nitrogen dynamics in the vegetation-soil-stream system, and the role of the riparian zone on nitrogen retention in near-stream zone.

**Koichiro Kuraji** is a Lecturer at The Tokyo University Forests, The University of Tokyo. He holds a Dr. Agric. degree from the University of Tokyo and is an Environmental Certified Public Measurer. Dr. Kuraji’s research interests include understanding the function of forests on flood, water resources and water quality, and its application to the integrated watershed management. In the University Forests, the effects of forest clear-cut and long-term forest re-growth on water yield and water quality is studied by paired watershed approach and 73 years continuous streamflow data, respectively. In Thailand, he is involved in the GAME-T project and upstream-downstream conflict management project in the Mae Chaem and Mae Tia watersheds, respectively. Dr. Kuraji also works in Lambir and Kinabalu National Park in Malaysian Borneo to study tropical forest meteorology and hydrology.

**Hiroto Toda** is an Associate Professor, Faculty of Agriculture at Tokyo University of Agriculture and Technology, where he earned his Ph.D. He was presented with the Japanese Forestry Society Progress Award in 1998 in the study of kinetics of nitrogen mineralization in forest soil, and the Japanese Society of Forest Environment Prize in 1999 in the study of characteristics of carbon mineralization in disturbed and undisturbed incubations of forest soil. His research focuses on nutrient circulation in forest ecosystem with special attention paid to the characteristics of nitrogen and carbon mineralization in forested soil. He has also been very interested in the effects of acid rain and high nitrogen deposition on forest ecosystems because little is known about their long-term effects on forest ecosystems in Japan. Dr. Toda has been researching the water chemistry and nutrient circulation in old growth Japanese cypress (*Chamaecyparis obtusa*) and cedar (*Cryptomeria japonica*) forested watersheds for over 20 years.

**Kaichiro Sasa**, Ph.D., is a Professor at the Field Science Center for Northern Biosphere, Hokkaido University. His current research interests are to clarify the historical changes and response for the environmental change of water and sediments movements, to clarify the buffering function of forest and watershed environment, and to develop the watershed managements and the tool for the appropriate land-use and forest utilization.
